# Recent advances in understanding and preventing human papillomavirus-related disease

**DOI:** 10.12688/f1000research.9701.1

**Published:** 2017-03-14

**Authors:** Karin Hellner, Lucy Dorrell

**Affiliations:** 1 Nuffield Department of Obstetrics and Gynaecology, University of Oxford, Women's Centre, John Radcliffe Hospital, Oxford, UK; 2 Nuffield Department of Medicine, University of Oxford, NDM Research Building, Old Road Campus, Headington, Oxford, UK; 3 Oxford NIHR Biomedical Research Centre, University of Oxford, The Joint Research Office, Block 60, Churchill Hospital, Old Road, Headington, Oxford, UK

**Keywords:** Human papillomavirus, cervical cancer, HPV lesions

## Abstract

High-risk human papillomaviruses (hrHPV) are responsible for anogenital and oropharyngeal cancers, which together account for at least 5% of cancers worldwide. Industrialised nations have benefitted from highly effective screening for the prevention of cervical cancer in recent decades, yet this vital intervention remains inaccessible to millions of women in low- and middle-income countries (LMICs), who bear the greatest burden of HPV disease. While there is an urgent need to increase investment in basic health infrastructure and rollout of prophylactic vaccination, there are now unprecedented opportunities to exploit recent scientific and technological advances in screening and treatment of pre-invasive hrHPV lesions and to adapt them for delivery at scale in resource-limited settings. In addition, non-surgical approaches to the treatment of cervical intraepithelial neoplasia and other hrHPV lesions are showing encouraging results in clinical trials of therapeutic vaccines and antiviral agents. Finally, the use of next-generation sequencing to characterise the vaginal microbial environment is beginning to shed light on host factors that may influence the natural history of HPV infections. In this article, we focus on recent advances in these areas and discuss their potential for impact on HPV disease.

## Introduction

Human papillomavirus (HPV) is the most common viral infection of the reproductive tract. Even though most HPV infections are asymptomatic and clear spontaneously, persistent infections with “high-risk” (oncogenic) mucosal HPV (hrHPV) cause approximately 5% of all cancers worldwide. These include almost all cases of cervical cancer––with annually over 500,000 newly diagnosed cases and over 260,000 cervical cancer deaths worldwide––as well as a large proportion of other anogenital carcinomas and oropharyngeal tumours in both women and men ^1^. The overall burden of HPV-related disease is difficult to estimate, but it is believed that approximately 600,000 annual cases of cervical, anal, penile, vulvar, and vaginal cancers combined are attributable to hrHPV ^2– 4^ ( Table 1).

**Table 1.** High-risk HPV-induced cancers.

Site	Percentage of cancers associated with high-risk HPV infection	Number of cancers attributable to high-risk HPV
Cervix	100	529,500
Anus	84	25,536
Vagina	70	10,500
Penis	47	12,361
Vulva	40	12,000
Oropharynx	19	11,685

Table modified from 3

**Figure 1.**
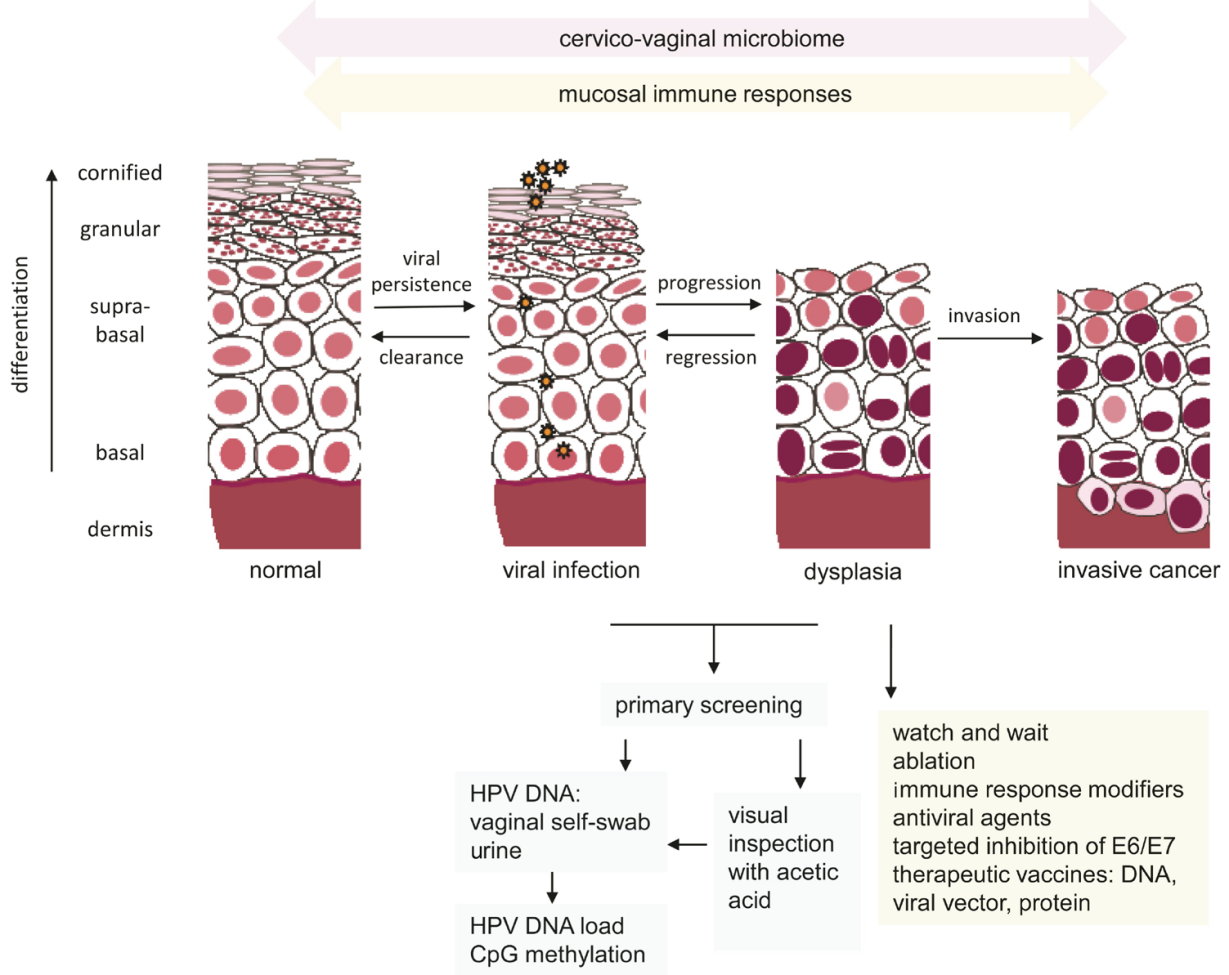
HrHPV-host interactions leading to invasive disease and strategies for intervention. Human papillomaviruses infect cells in the basal layer of squamous epithelia through sites of microtrauma. Infection with high-risk HPV (hrHPV) usually clears spontaneously but persistent infection can lead to intraepithelial neoplasia and rarely, invasive cancer (adapted from 2). Effective innate and adaptive immune responses may lead to regression of dysplastic lesions and clearance of infection. Changes in the cervico-vaginal microbiome have also been implicated in acquisition and clearance of hrHPV infection. Technological advances discussed in this review that have been shown to improve screening and treatment of hrHPV disease are listed.

Cervical cancer is preventable. In the developed world, women who die from cervical cancer either have had little or no screening throughout their lifetime or have not accessed appropriate treatment for abnormal cytology, hence it is not surprising that the majority (around 85%) of the global burden occurs in the less-developed regions with little or no access to screening or prophylactic vaccine programmes ^5^. An additional concern is that the HIV epidemic has disproportionately affected the same regions and is an important contributing factor to the prevalence and persistence of HPV infections ^6^. Closing the gap between wealthy and low-income countries in prophylactic HPV vaccine rollout is an international priority. However, even if this were to be achieved in the near future, hundreds of millions of women who have already been exposed to hrHPV will remain at risk of developing cervical cancer without access to affordable tools for screening and treatment of pre-invasive disease. In this article, we review innovations in testing and treatment for hrHPV infection and intraepithelial neoplasias from molecular, clinical, and operational perspectives alongside new insights into pathogenesis (summarised in Figure 1).

## Advances in screening

HPV prevalence varies largely by geographic region, age, gender, and sexual behaviour. HPV-16 antibody prevalence in females aged 9–26 ranged from 0–33% in Europe and North America to 13–43% in Africa and Central and South America ^7^. HrHPV-16 and -18 DNA prevalence was consistently lower than antibody prevalence; however, rates varied widely according to age. In a study assessing the impact of prophylactic vaccination in England, prevalence of hrHPV DNA (any of 13 types) in women aged 16–24 during 2012–2013 ranged from 35–44% ^8^. Although rare, the presence of antibodies against hrHPV in prepubertal children may suggest non-sexual (perinatal or horizontal) transmission routes ^7, 9, 10^. HPV-16 and -18 cause nearly 70% of cervical cancers. HPV-16 also contributes largely to hrHPV-associated vulvar, vaginal, and penile pre-cancer ^11^.

For the past five decades, the introduction of screening with the Papanicolaou (Pap) smear test to detect cervical disease early and the implementation of national screening programs have reduced the incidence and mortality of a disease that once caused more deaths amongst women than any other cancer. Liquid-based cytology has widely replaced the conventional Pap testing in recent years, owing to its greater effectiveness, i.e. comparable sensitivity coupled with reduced risk of inadequate sampling ^12^. However, in the absence of hrHPV testing, high false-positive detection rates can lead to unnecessary referrals to colposcopy. This triggered the recent use of hrHPV DNA testing to triage women with abnormal cytology at screening. Although this is standard of preventive care in high-income countries, access to screening in LMICs is still limited or unavailable. Population-based models have calculated a projected reduction in cervical cancer incidence of 50–60% by 2040 if effective screening and/or treatment methods were to be implemented ^13^. Fortunately, the growing importance of reducing the burden of HPV-induced cancers has been recently acknowledged by the World Health Organisation and led to the listing of screening and treatment of precancerous lesions to prevent cervical cancer as a “best buy” intervention ^14^.

HrHPV testing detects over 90% of all high-grade cervical intraepithelial neoplasia (CIN) and is therefore an attractive screening tool, especially in countries with established screening infrastructure. Several countries are due to implement primary hrHPV testing with triage by cytology or, in some settings, partial genotyping ^15^. Large randomised controlled trials have shown that hrHPV testing, when implemented over several rounds of screening, provided greater protection against CIN2+ or invasive cervical cancer than did cytological screening. Of note, protection was greater with hrHPV screening in women at 30+ years of age and with a screening interval of 5 years than cytological screening every 3 years ^16– 19^. Even a single round of HPV testing can have a significant impact on the rate of advanced cervical cancers and cancer deaths in rural India ^20^. Currently, the implementation of molecular testing in LMICs is limited because of cost and infrastructure requirements ^15^. However, this might be addressed by using rapid low-cost point-of-care tests such as Xpert HPV, which has shown promise in a field trial in Papua New Guinea ^21^.

HPV self-testing to collect cervical/vaginal cells offers the potential to improve screening coverage: meta-analyses have concluded that self-testing increased the participation of non-attenders in screening programmes when sampling kits were provided directly to all participants ^22^. However, the effectiveness of screening by self-sampling is determined in part by the performance of the HPV PCR test when applied to self-samples ^23^. To bridge the prevention gap in low-/middle- income settings, approaches such as hrHPV self-testing are viable and enable invitation of communities who test positive to attend further triaging ^24, 25^. Visual inspection of the cervix with acetic acid (VIA) or Lugol’s iodine (VILI) is a particularly simple and low-cost screening tool that enables trained healthcare workers to detect abnormalities with a speculum examination. Both methods are especially attractive tools for establishing screening in LMICs, where the infrastructure to provide screening at a national level is not yet in place despite increasing awareness of its importance ^26– 28^. HPV urine testing is a non-invasive alternative to conventional cytology sampling that could improve screening uptake in individuals for whom cervical or vaginal sampling may be difficult. A large meta-analysis confirmed that detection HPV test results in urine generally correlate with HPV testing in clinician-taken samples ^29, 30^. However, data to support urinary HPV testing for the detection of CIN are currently lacking.

HrHPV genotypes are maintained in the general population owing to productive infections rather than inadvertent cancers. Low-grade lesions (CIN1), where infectious particles are produced and shed, tend to regress spontaneously within 18–36 months in immunocompetent hosts ^31^. Whether a productive life cycle is completed or not depends on the infected epithelial site and the hormonal environment ^32^. Nevertheless, hrHPV can persist––often for many years––and can drive cell cycle entry and cell proliferation in the basal and parabasal cell layers ^33^, which distinguishes them from low-risk HPVs. Progression to cancer is a consequence of persistent hrHPV infection that, following integration of the viral genome into host DNA, drives dysregulated viral gene expression. The hrHPV early proteins E6 and E7 inactivate the host tumour suppressors p53 and retinoblastoma protein, resulting in neoplastic transformation. The distinct susceptibility of the transformation zone to neoplastic transformation and progression may also be linked to the increased accessibility and proliferation of the basal cell layers at this metaplastic epithelium ^33^.

Recently, the predictive value of HPV viral load as a measure of persistence has been investigated: several studies have shown that high baseline HPV-16 and -18 viral load or viral load increase over time are associated with persistent infections ^34– 36^ and low viral load, or a >100-fold decline over time, is associated with clearance ^37– 39^. Higher viral load was also associated with decreased HPV clearance rates in uncircumcised males and homosexual men who smoke ^40, 41^. Methylation of CpG sites in HPV DNA is a marker of persistent infection, particularly for HPV-16, that may have utility in triage: in a large randomised controlled trial, methylation triage on cervicovaginal self samples was non-inferior to cytology triage on clinician-taken samples for the detection of high-grade CIN ^42^.

## Advances in therapy of HPV-associated disease

Precancerous cervical lesions can be treated by loop excision of the transformation zone or ablative techniques such as cryotherapy or laser therapy. The use of novel, cheap ablation techniques that do not require external gas supply (such as CryoPen) could provide affordable treatment of precancerous lesions and prevent progression to cancer in LMICs ^43^. Recently, more conservative approaches in the management of moderate (CIN2) lesions have been adopted in the light of large population-based data indicating high regression rates in young, non-smoking, immunocompetent women in whom minimising the risk of future adverse pregnancy outcomes (second-trimester miscarriage and spontaneous pre-term delivery) is a priority ^44– 47^.

A non-surgical therapy for pre-invasive hrHPV lesions is highly desirable given the disadvantages outlined above and the infrastructure required to deliver and monitor the efficacy of ablative treatment, which is a major challenge for LMICs. In addition, there is a need for better therapeutic options for hrHPV-driven lesions at anogenital sites other than the cervix. Tackling chronic and recurrent HPV-induced lesions and the possibility to scale up patient-applied use in LMICs have led to the development of the topical immune response modulators such as imiquimod and Yallaferon®, a recombinant interferon alpha-2b gel, but recurrence rates remain high. Lopinavir and cidofovir, both antiviral drugs, are currently being trialled in precancerous lesions as well as HPV-associated cancers of varying sites ^48, 49^. Multiple small molecule inhibitors targeting hrHPV DNA-binding activities or the apoptotic sequences of E6/E7 or exhibiting synthetic lethal interactions are still in preclinical development ^50^. Adoptive T cell therapy comprising infusion of E6- and E7-specific tumour-infiltrating T lymphocytes to facilitate tumour shrinkage is a novel approach to the treatment of metastatic cervical cancer that has shown promise in an exploratory clinical study ^51^. However, such individualised therapies are challenging to deliver at scale.

Several aspects of hrHPV biology and pathogenesis make it an attractive candidate for targeting with a therapeutic vaccine. The development of a high-grade cervical lesion is the result of failure of host T cell responses to control or indeed clear hrHPV infection, an uncommon event that typically takes 10–15 years and is reversible in up to 30% of cases ^31, 52– 54^. Regression of CIN is associated with infiltration of the lesion by CD8+ T cells ^55^. CD4+ T cells play a crucial role in orchestrating this effector response: immunosuppressive states (e.g. HIV infection, iatrogenic) are associated with reduced HPV clearance rates and increased risk of progression to invasive cancer ^56– 59^. T cells recognise viral antigens in the form of peptides that are generated from proteolytically cleaved internal and external proteins and presented on the cell surface in association with HLA class I (CD8+ T cells) and class II (CD4+ T cells) molecules. It is hypothesised that boosting naturally induced hrHPV-specific T cell responses to early viral antigens by vaccination should accelerate the regression of CIN and clearance of infection. Licensed prophylactic vaccines prevent the acquisition of infection through the induction of antibodies to late (capsid) proteins presented in the form of virus-like particles. They have no impact on disease once infection has occurred; therefore, a different vaccine strategy is required to achieve a therapeutic effect. HPV (along with other oncogenic viruses) presents non-self antigens; the development of T cell tolerance is therefore far less likely than is the case for cancers expressing self-antigens. The oncoproteins E6 and E7 have been the antigens of choice for most therapeutic vaccine candidates to date, as they are expressed throughout the virus life cycle and on transformed cells. The development of therapeutic vaccines for hrHPV has encompassed proteins and peptides, viral, bacterial, and DNA vectors, RNA replicons, and dendritic cell-based approaches. As there have been several recent comprehensive reviews ^60, 61^, we focus on the most clinically advanced strategies.

Peptide- and protein-based vaccines offer safety, stability, and ease of manufacture. However, they are poorly immunogenic unless administered with adjuvants, which may cause unwanted reactogenicity, and efforts to apply this approach to immunotherapy of hrHPV have generally been unsuccessful. A mix of adjuvanted synthetic long peptides from HPV-16 E6 and E7 was found to induce complete regression in 9 out of 19 women with high-grade vulval intraepithelial neoplasia ^62^. This was an important finding given the very low rate of spontaneous regression of vulval intraepithelial neoplasia and high recurrence rate. A randomised placebo-controlled phase II trial was subsequently initiated to assess the same vaccine strategy in women with HPV-16-positive high-grade CIN. Unfortunately, the study was terminated prematurely because of the reluctance of enrolled patients to defer excisional treatment; therefore, the results were inconclusive ^63^. A recombinant protein vaccine, GTL001, comprising HPV-16 and -18 E7 fused to catalytically inactive *Bordetella pertussis* adenylate cyclase, was tested alone and with imiquimod in a phase I study of women with HPV-16 or HPV-18 and normal cytology ^64^. Sustained viral clearance was observed in the majority of women receiving the higher vaccine dose together with imiquimod.

DNA vaccines are safe and easy to manufacture, do not require a cold chain, and can be administered repeatedly, as they do not elicit vector-specific immunity. However, despite eliciting potent immune responses in small animal models, immunogenicity in humans has been disappointing. Electroporator delivery and adjuvants can overcome this, albeit at the cost of increasing complexity and reactogenicity. The leading candidate is the VGX-3100 vaccine (Inovio Pharmaceuticals), which comprises synthetic consensus HPV-16 and HPV-18 E6 and E7 genes. It has shown the most encouraging results of any therapeutic HPV vaccine candidate to date in a randomised controlled phase IIb trial in 154 women with CIN2/3 ^65^. In the modified intention-to-treat analysis, histopathological regression to CIN1 or normal was significantly more frequent in VGX-3100 recipients than in placebos (48.2% vs. 30%). In a post-hoc analysis, the magnitude of E6-specific T cell responses was associated with regression of CIN and viral clearance ^65^. A similar DNA vaccine expressing E6/E7, GX-188E, was tested in a small uncontrolled study: complete regression and virus clearance was observed in seven out of nine women with CIN3, in association with polyfunctional CD8+ T cell responses ^66^.

Viral vector vaccines elicit potent cell-mediated immune responses owing to their capacity for high levels of transgene expression and natural adjuvant properties. Heterologous prime-boost viral vector regimens targeting diverse human pathogens have been shown to induce high frequencies of T cells in clinical trials while avoiding vector-specific immunity ^67^. The first trial of a replication-competent vaccinia virus vectored HPV vaccine, TA-HPV, was conducted 20 years ago ^68^. A modified vaccinia Ankara (MVA) vectored vaccine encoding E2, a transcriptional regulator of E6 and E7, has been tested in a phase III trial that included 1,176 female subjects with HPV-driven oncogenic and non-oncogenic intraepithelial lesions and 180 males with condylomata only ^69^. The vaccine was delivered directly into the affected anogenital tissues. The investigators reported elimination rates of 89% in females and 100% in males. When considering only the participants with high-grade lesions (300/1,176 women), the cure rate was 73% ^69^. Although this is higher than the expected spontaneous regression rate, the results should be interpreted with caution, as a control group was lacking.

## Role of the cervico-vaginal microbiome in the development of cervical cancer

HrHPV infections are highly prevalent in sexually active women, yet only a small minority persist and progress to cancer. Reported risk factors for carcinogenesis include smoking, other sexually transmitted pathogens, oral contraceptive use, and socio-economic status. However, few studies have established which of these associations have a mechanistic basis and which are confounders reflecting high-risk sexual behaviour. Recent studies using next-generation sequencing to characterise the microbial communities inhabiting the vagina and cervix (the cervico-vaginal microbiome) have suggested a possible role of altered vaginal microbiota in the development of CIN and cervical cancer. The cervico-vaginal microbiome is typically of low diversity and dominated by Lactobacillus species, which are assumed to protect against pathogens by maintaining a low pH ^70, 71^. Vaginal dysbiosis (bacterial vaginosis [BV]), a state characterised by paucity or absence of lactobacilli, overgrowth of anaerobes, and high pH, has been implicated in the acquisition and outcome of HPV infections. A systematic review and meta-analysis of 19 studies confirmed an association between BV and CIN ^72^. However, causality remains uncertain, since the majority of studies were cross-sectional. A reduction in Lactobacilli, increasing vaginal microbial diversity, and dominance of Sneathia species were observed in association with progression of cervical histology from normal through to high-grade CIN ^73, 74^. In a longitudinal study in which women were sampled twice weekly for 16 weeks, community state types (CSTs, distinct clusters of vaginal bacterial species) were observed to influence both the incidence and the clearance rates of prevalent HPV infection ^75^. A low lactobacillus CST was associated with a nearly two-fold higher rate ratio of incident HPV, and HPV clearance was fastest in women with *Lactobacillus gasseri*-dominated CSTs.

To date, the microbial composition in the vagina and cervix of healthy women has been studied largely through parallel sequencing of the highly conserved 16s ribosomal RNA subunit genes, which indicates the abundance and diversity of bacteria but does not provide information on the functional activity of the human vaginal microbiota or the diversity of other microbes present. Direct DNA sequencing (metagenomics) has revealed not only a very high prevalence of HPV in healthy women but also a far greater diversity of HPV types than can be detected by conventional typing ^76^. Metatranscriptomics approaches can yield more precise information regarding function and thus identify potential targets, such as the metabolically active bacteria in dysbiotic states ^77^. A possible interaction between cervical microbiota diversity, histopathological diagnosis of cervical HPV lesion, and cervical cytokine expression was explored in a recent cross-sectional study of women across a spectrum of HPV disease. Expression of IL-4, IL-10, and TGF-β was increased in women with cervical cancer compared with those with low-grade CIN ^74^. It was hypothesised that hrHPV E2, E6, and E7 drive the production of immunosuppressive cytokines, which, in turn, increase cervical microbial diversity and proliferation of Sneathia and Fusobacterium species. Collectively, these studies highlight the need for more longitudinal data and for a combined metagenomics/metatranscriptomics approach in order to better understand the complex interplay between the cervico-vaginal microbiota, sexually transmitted pathogens, and host immune system in determining the outcome of hrHPV infections.

## Looking forward and challenges to implementation

Implementation of effective cervical screening is a major medical advance of the late twentieth century, and it will remain an essential tool for cancer prevention for several decades while prophylactic HPV vaccination programmes take effect. The transition from cytology to hrHPV detection as a screening tool has the potential to improve the effectiveness of screening at longer intervals and to increase coverage by enabling self-testing in the community or even at home. Stratification according to age and HR genotype, with fast-tracking of HPV-16-positive cases to colposcopy and treatment could reduce unnecessary referrals and healthcare costs in high-income countries and also facilitate the allocation of limited resources in LMICs. The opportunities available to LMICs through technological innovations in screening and preventive treatment will be missed unless innovative approaches to implementation are adopted. These include “screen and treat” protocols that embed screening by VIA or HPV tests in existing primary healthcare systems, scaling up training of healthcare workers to enable provision of treatment in a greater range of settings than specialised cancer services, and use of smartphone technology for tracking, recalls, and staff training ^78– 80^. The use of a low-cost ultra-portable tampon-based digital colposcope has been proposed as a novel approach to increase access to screening at the community level and also as a tool for virtual training ^81^. Collectively, these efforts could have significant health and socio-economic benefits.

Although the development of specific antiviral agents is slow, alternatives to surgical excision or ablation of CIN such as therapeutic DNA vaccines are on the horizon. With the application of potent viral vector vaccine technologies, we anticipate improvements in the clinical efficacy of these approaches. As our understanding of the microbial and mucosal immune states that lead to persistent hrHPV infection grows, we may identify new agents that can be used to manipulate the local microenvironment for therapeutic benefit.
